# Identification of a novel alphavirus related to the encephalitis complexes circulating in southern Brazil

**DOI:** 10.1080/22221751.2019.1632152

**Published:** 2019-06-25

**Authors:** Marcel Kruchelski Tschá, Andreia Akemi Suzukawa, Tiago Gräf, Laercio Dante Stein Piancini, Allan Martins da Silva, Helisson Faoro, Irina Nastassja Riediger, Lia Carolina Medeiros, Pryscilla Fanini Wowk, Camila Zanluca, Claudia Nunes Duarte dos Santos

**Affiliations:** a Laboratório de Virologia Molecular, Instituto Carlos Chagas/Fiocruz PR, Curitiba, Brazil; b Departamento de Genética, Instituto de Biologia, Universidade Federal do Rio de Janeiro, Rio de Janeiro, Brazil; c Laboratório Central, Secretaria da Saúde do Estado do Paraná, São José dos Pinhais, Brazil; d Laboratório de Regulação da Expressão Gênica, Instituto Carlos Chagas/Fiocruz PR, Curitiba, Brazil; e Laboratório de Biologia Celular, Instituto Carlos Chagas/Fiocruz PR, Curitiba, Brazil

**Keywords:** Caainguá virus, *Culex*-associated virus, mosquito, new alphavirus, South Brazil

## Abstract

In early 2017, an outbreak caused by an unknown and supposedly viral agent in the Marilena region of southern Brazil was investigated. Since the etiological agent causing the outbreak was not identified from human samples, mosquitoes from this region were collected. Three out of 121 mosquito pools collected from the region tested positive for alphavirus in molecular tests. Next generation sequencing results revealed the presence of a novel alphavirus, tentatively named here as Caainguá virus (CAAV). DNA barcoding analyses indicated that different species of *Culex* are hosts for CAAV. This new virus was basal to the New World encephalitic alphaviruses in a comprehensive and robust phylogenetic approach using complete genomes. Viral particles were observed in the cytosol and inside of intracellular compartments of cells in mosquito-derived cell cultures. Despite being noninfectious in vertebrate derived cell cultures, primary culturing of CAAV in human mononuclear cells suggests monocytes and lymphocytes as CAAV targets. However, the epidemiological link of CAAV on the human outbreak should be further explored.

## Introduction

The genus *Alphavirus* of the *Togaviridae* family comprises enveloped, single-stranded positive-sense RNA viruses and currently includes over 30 species [1]. Members of this genus have an ∼11.7 Kb genome with a capped 5′ end and a poly-A tail at the 3′ end and is divided into two open reading frames, the nonstructural and the structural domains [2]. Most alphaviruses are mosquito-borne viruses and have a wide range of vertebrate hosts, including humans [2]. Recently, with advances in viral detection methodologies using next generation sequencing and bioinformatics tools, a growing number of novel viruses have been described in *Togaviridae* family, including viruses with unidentified vertebrate hosts [3–5] and those with fishes as natural hosts [6]. Humans infected by pathogenic alphaviruses exhibit febrile illnesses that may culminate either in encephalitis or arthritis, depending upon the viral etiology. Alphaviruses are broadly distributed on all continents and are primarily transmitted to humans by female mosquitoes during blood meal feeding. These viruses are classically divided into Old World and New World viruses [7], with Old World viral infections often causing clinical symptoms such as fever, rash and arthritis, whereas New World viral infections are associated with encephalitis. However, the recent spread of Chikungunya virus (CHIKV), originally from the Old World, to the Americas [8–9], demonstrates the dissemination dynamics of arboviruses worldwide.

In the first half of 2017, the Reference Laboratory of Emerging Viruses from Fiocruz-PR received sixty-two serum samples from patients who presented to the health service with fever, myalgia, headache, backache, retroorbital pain, and arthralgia. Ten of the serum samples were collected during the acute phase of disease (between 1 and 6 days after the onset of symptoms). These acute phase serum samples were screened for infection by flavivirus and alphavirus with generic RT–PCR protocols, as well as for MAYV, WNV, and OROV using specific primers, and all rendered negative results. All of the patients were inhabitants of Marilena municipality (22° 44′ 09″ S, 53° 02′ 24″ W), Paraná State, Southern Brazil. As the etiological agent causing this outbreak was not identified, mosquito samplings were performed in the field and near the houses of the patients.

**Figure 1.**
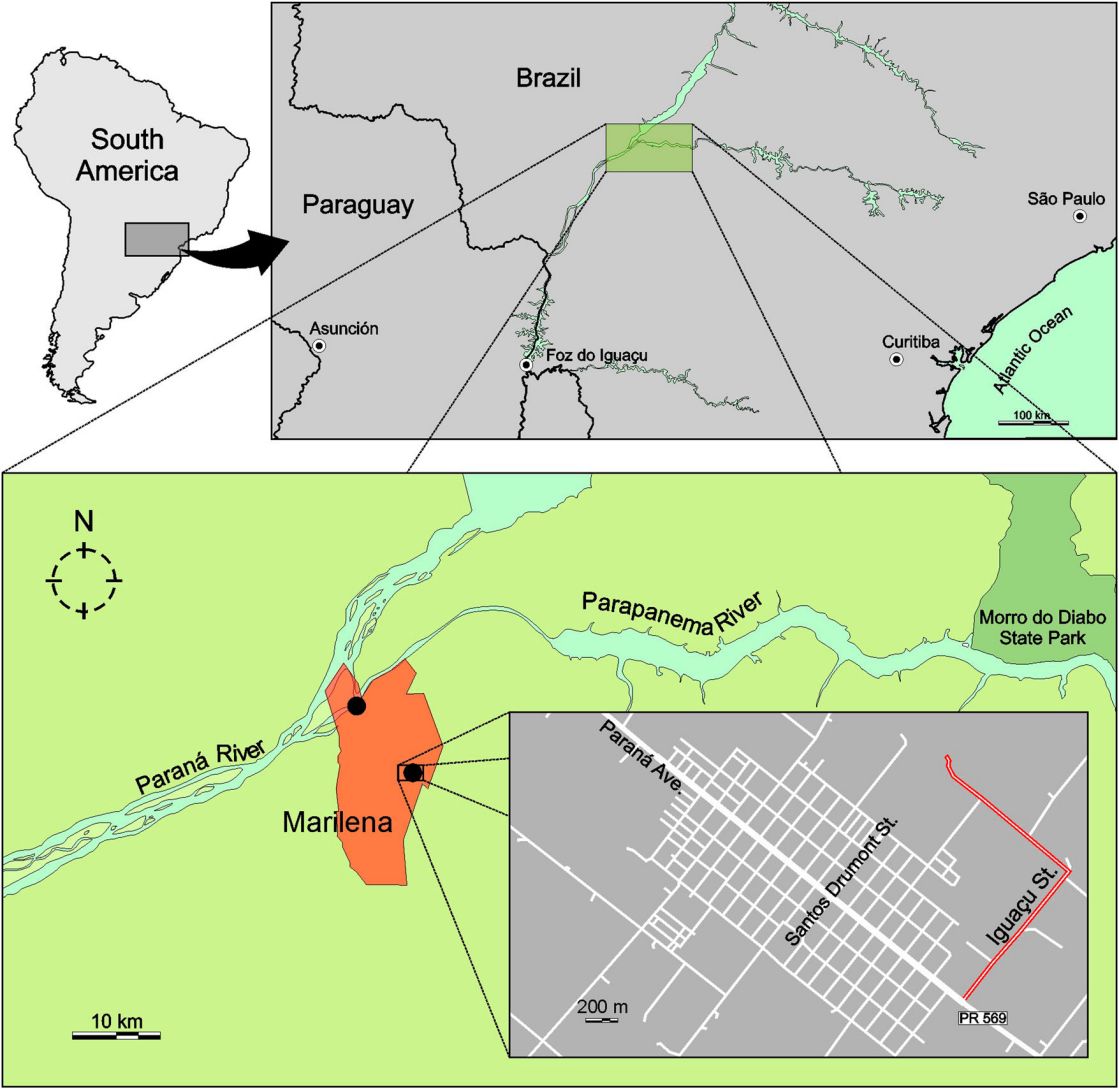
Sampling site of CAAV positive pools of mosquitoes (black dots) and the location of Marilena municipality, State of Paraná, Brazil. In the last frame, the county detail with the place of the collection of pool code MS364 is highlighted in red. Pool codes MS680 and MS681 were collected in the margin of Paraná River.

Marilena is a city with approximately 7,000 inhabitants located at the left margin (downstream) of the Paraná River in confluence with Paranapanema River (Figure 1). This region is at a low altitude and has a humid subtropical climate zone according the Köppen-Geiger climate classification map [updated in ref. 10]. In the past few years, this region has presented a high incidence of arbovirus circulation, primarily dengue virus [11]. Nevertheless, during the epidemiological period 2016/2017, approximately 88% of the patients seeking health care services with clinical symptoms of a dengue-like disease had no laboratory confirmed diagnosis of dengue [12], thus constituting an interesting context to perform prospection of new viruses.

This study describes a novel alphavirus, tentatively named Caainguá virus (CAAV), that was isolated from pools of *Culex* mosquitoes collected at Marilena municipality, Brazil, during an outbreak of an undiagnosed arthritogenic disease. Here we present the phylogenetic analysis as well as a biological characterization of CAAV, including viral replication in mosquito, vertebrate and primary human cells and electron microscopy. So far, the results suggest the discovery of a new insect-specific alphavirus circulating during an undiagnosed human outbreak scenario. Nevertheless, we cannot rule out an epidemiological link between CAAV and the human cases due to the intriguing property of CAAV to infect human primary cells.

## Materials and methods

### Sampling of mosquitoes and molecular screening

Mosquito collection was performed by the Epidemiological Surveillance group of the Paraná State Health Service, concomitant with the ongoing human outbreak of the unknown disease (May and December 2017). Adult mosquitoes were collected using insect aspirators and by active collection techniques between 9:00 am and 4:00 pm and were pooled according to morphological identification, sampling location and date. Morphological identification was performed according to Lane and Forattini [13,14].

Briefly, mosquitoes were mechanically lysed in RNase-free phosphate buffered saline (PBS) using a Precellys^TM^ homogenizer (Bertin Instruments, Montigny-le-Bretonneux, France). RNA was extracted using a QIAamp Viral RNA Mini Kit^TM^ (Qiagen, Hilden, Germany) according to the manufacturer's protocol, and the remaining homogenates were stored at −80°C. A volume of 5.6 µL of the extracted RNA was used in the cDNA synthesis step. Alphavirus prospection was performed using generic primers described by Sánchez-Seco et al. [15]. Nested PCR was performed in a final volume of 25 µL. The first-round PCR was performed following an initial denaturation of 2 min at 94°C followed by 40 cycles: 30 s at 94°C, 60 s at 52°C, and 60 s at 72°C, with a final extension at 72°C for 5 min. The PCR reaction contained 2.5 U of Platinum Taq DNA polymerase (Thermo Fisher Scientific, Waltham, USA), 1× buffer, 2.5 mM MgCl_2_, 0.1 mM of each dNTP, 20 pmol of each primer, and 5 µL of cDNA. The second-round PCR was performed following an initial denaturation of 2 min at 94°C followed by forty cycles: 30 s at 94°C, 30 s at 52°C, and 30 s at 72°C, with a final extension at 72°C for 5 min. The PCR reaction contained 2.5 U of Platinum Taq DNA polymerase, 1× buffer, 2.5 mM MgCl_2_, 0.1 mM of each dNTP, 20 pmol of each primer, and 1 µL of the first-round PCR product. PCR products were visualized on an 1.5% agarose gel stained with ethidium bromide. Mosquito pools were also screened for flavivirus infection according to the protocol described by Sánchez-Seco et al. [16].

### CAAV isolation and titration

Viral isolation from the *Culex* mosquito MS681 pool lysate was performed in C6/36 cells (derived from *Aedes albopictus* larvae) (ATCC® CRL-1660; Manassas, USA). Cells were grown in Leibovitz's L-15 medium (Gibco, Waltham, USA) supplemented with 5% foetal bovine serum (FBS) (Gibco), 0.26% tryptose (Sigma-Aldrich, St. Louis, USA) and 25 µg/mL gentamicin (Gibco) at 28°C. No cytopathic effect was observed until 14 d.p.i. A subsequent passage was performed (passage 1), in which viral isolation was confirmed by RT–PCR and immunofluorescence assay (IFA).

A foci-forming immunodetection assay in C6/36 cells was used for viral titration [17]. Briefly, tenfold dilutions of viral supernatants were added to cell monolayers followed by a 1-hour incubation at 28°C. After absorption, the inoculum was removed and 500 µL of overlay (composed of 1,6% carboxymethyl cellulose and L-15 medium supplemented with 5% FBS, 0.26% tryptose and 25 μg/mL of gentamicin) were added to each well. The plates were incubated at 28°C for 7 days. After discarding the overlay, the cells were fixed with 3% paraformaldehyde and permeabilized with 0.5% Triton X-100. Anti-Alphavirus E1 protein (clone 1A4B.6, cat. MAB8754, Merck, Temecula, USA) and anti-mouse IgG AP conjugate (Promega, Madison, USA) were used as the primary and secondary antibodies, respectively. Finally, foci were revealed by the addition of NBT-BCIP substrate (Promega, Madison, USA), and the titre was expressed in focus-forming unit (ffu/mL).

### Viral genome characterization and phylogenetics analyses

Genomic RNA derived from the isolated virus was amplified and sequenced using a combination of the generic primers Alpha1+ from the protocol of Sánchez-Seco et al. [15] and Pan-Alpha-R1 from Hermanns et al. [4] to increase the fragment length. The PCR products were purified using a High Pure PCR Product Purification Kit (Roche, Mannheim, Germany) before sequencing.

Whole-genome sequencing of the isolated virus was performed to confirm the identity of the novel virus. RNA purification was performed using a QIAamp Viral RNA Mini Kit (Qiagen) following the manufacturer's instructions, excluding the use of the RNA carrier reagent to facilitate sequencing procedures. Purified RNA was used for library construction using a TruSeq RNA kit (Illumina, San Diego, USA) and was sequenced on an Illumina MiSeq platform (2 × 75 bp). The obtained reads were uploaded to CLC Genomics Workbench v.10.5 (Qiagen) and assembled using the *De novo assembly* pipeline in the default configuration.

To understand the evolutionary relationship of the virus being described in this study, phylogenetic and recombination analyses were performed. After genome sequencing, the generated consensus sequence was aligned with representative sequences for all alphavirus species, its variants and subtypes described so far. Up to three sequences per virus species were downloaded from GenBank (http://ncbi.nlm.nih.gov/genbank/) depending upon availability. Nucleotide sequences were codon aligned using MACSE v2.03 [18], and ambiguously aligned portions were cleaned using Gblocks v. 0.91b [19]. In addition to the full genome sequences, with fragments of two open reading frames concatenated, alignments encompassing the alphavirus nsP and the complete sP regions were generated and analysed separately (results in supplementary material).

Phylogenetic analyses were performed using the Maximum-likelihood (ML) and Bayesian approaches. ML trees were inferred using IQ-TREE, and branch support was calculated using ultrafast bootstrap approximation (UFBoot) with 1000 replicates [20]. Prior to the tree reconstruction, the ModelFinder application [21], as implemented in IQ-TREE, was used to select the best-fitted nucleotide substitution model. Bayesian trees were estimated using MrBayes v3.2.7 [22], where the nucleotide substitution model was set according to the ModelFinder results. For each dataset, two runs of four chains each (one cold and tree heated) were run for 3.8–10 × 10^6^ generations, with a burn-in of 25%. All parameters estimated for each run showed ESS values above 200, and a final Bayesian majority-rule consensus tree was summarized for each dataset. Finally, the full-length genome alignment was screened for the presence of recombination breakpoints using the GARD algorithm from the HyPhy package v2.3.13 [23] and bootscanning analysis, as implemented in Simplot 3.5.1 [24]. A window size of 1000 bp with a step of 50 bp was used for bootscanning, and sequences were grouped into their respective complexes when appropriate.

### DNA barcoding of CAAV hosts

To confirm the mosquito species identity in alphavirus-positive pools, fragments of the cytochrome C oxidase subunit I (COI) mitochondrial gene were amplified using two sets of genetic markers: the primer pair LCO1490 and HCO2198 described by Folmer et al. [25] and the primer pair F-COI50 and R-COI650 described by Hemmerter et al. [26]. Initially, insect genetic material was obtained from mosquito homogenates using a Genomic DNA Extraction Kit (RBC Real Genomics^TM^, Banqiao City, Taiwan). PCR was performed using an initial denaturation of 3 min at 95°C followed by thirty five cycles: 30 s at 95°C, 30 s at 48°C, and 45 s at 72°C, with a final extension at 72°C for 5 min. PCR reactions contained 2.5 U of Taq DNA polymerase, 1 × PCR buffer, 2 mM MgCl_2_, 0.4 mM dNTP, 1 µM of each of the forward and reverse primers, and 1 µL of template DNA in a final volume of 25 µL. PCR product was purified using a High Pure PCR Product Purification Kit before conventional Sanger sequencing.

### Transmission electron microscopy

C6/36 cells were infected with CAAV or CHIKV BR/2015/15010 (isolated from a Brazilian human case in 2015, used as positive control) at a MOI of 1 for 72 h. Mock or infected C6/36 cells were fixed (2.5% glutaraldehyde and 4% paraformaldehyde in 0.1 M sodium cacodylate buffer, pH 7.2) at room temperature for one hour. After washing twice with 0.1 M cacodylate buffer, cells were fixed in 1% OsO_4_, 0.8% KFe(CN)_6_ and 5 mM CaCl_2_ diluted in 0.1 M cacodylate buffer at room temperature for 45 min. Cells were washed twice with 0.1 M cacodylate buffer, dehydrated in increasing concentrations of acetone, and embedded in Poly/Bed 812 resin for 72 h at 60°C. Ultrathin sections (60 nm) were collected in copper grids, stained for 45 min with uranyl acetate and for 1 min with lead citrate. Subsequently, the samples were observed in a JEOL JEM-1400 transmission electron microscope operating at 90 keV.

### Infection of mosquito and vertebrate cells lines

The arthropod-derived cell lines Aag-2 (ATCC® CCL-125™, derived from *Aedes aegypti* larvae), AP-61 (derived from *Aedes pseudoscutellaris* larvae), and C6/36 (ATCC® CRL-1660; derived from *Aedes albopictus* larvae) were either cultured in Schneider's Drosophila Medium (Aag-2) or Leibovitz's L-15 (AP-61 and C6/36) and were maintained at 28°C. The vertebrate cell lines used in this study included Vero E6 (Sigma-Aldrich, 85020206, epithelial cells derived from *Cercopithecus aethiops* kidney), UMNSAH/DF-1 (ATCC® CRL-12203™, *Gallus gallus* embryo fibroblasts lineage), BHK-21 (ATCC® CCL-10™, baby *Mesocricetus auratus* kidney fibroblasts line), ZEM-2S (ATCC® CRL-2147™, *Danio rerio* embryo fibroblast line), Huh7.5 (ATCC® PTA-8561™, human hepatocellular carcinoma cell line), C6 (ATCC® CCL-107™, *Rattus norvegicus* glial cells) and A549 cells (ATCC® CCL-185, human lung epithelial cells), all of which were cultivated in the appropriate growth medium at 28°C (ZEM-2S) or 37°C with 5% CO_2_. Cells were infected with CAAV at MOIs of 1 or 10 for one hour. Venezuelan Equine Encephalitis Virus (VEEV) TC83 was used as positive control for UMNSAH/DF-1 and ZEM-2S cells, and CHIKV BR/2015/15010 was the positive control for the other cell lines. Subsequently, to avoid any interference of the remaining virus in the inoculum, the cell monolayers were washed three times with PBS before the addition of the appropriate media. Viral replication was evaluated 0, 24 and 72 h.p.i by IFA using commercial anti-alphavirus antibody (cat. MAB8754, Merck). Images were obtained with a Leica AF6000 Modular System with a 40× objective. Images were acquired with different exposition times to evidence the infected cells, without interfering with the percentage of positive cells.

### Primary human cell infection

Peripheral blood samples from six healthy adult donors, with ages ranging between 22 and 40 years and with no history of arbovirus infection, were collected after obtaining written consent (Human Research Ethics Committee from Fiocruz under the number CAAE: 60643816.6.0000.5248). Peripheral blood mononuclear cells (PBMCs) were obtained by density gradient separation with Ficoll-Paque PLUS (density 1.077 g/mL) (GE Life Science). Cells (5 × 10^5^/well, 96 well plate) were infected with CAAV or CHIKV BR/2015/15010 (positive control) at MOIs of 1 or 10 for one hour. After removing the viral inoculum, the cells were washed twice with PBS and PBMCs were maintained at 37°C with 5% CO_2_ in RPMI 1640 medium (Lonza) supplemented with 100 IU/ml penicillin (Gibco), 100 g/ml streptomycin (Gibco), and 10% FBS. Uninfected C6/36 cell supernatants were used as a mock control. Supernatants were recovered at 24, 48 and 72 h.p.i. and stored at −80°C for viral titration, and the cells were used for flow cytometry analysis. Briefly, the cells were stained with a mix of anti-human monoclonal antibodies (mAb) for surface markers, including anti-CD3-APC (Immunotools), anti-CD4-APC-H7 (BD Biosciences), anti-CD8-PE-Cy5 (BD Biosciences), anti-CD19-PE-Cy7 (BD Biosciences), and anti-CD14-BV450 (BD Biosciences), or their respective isotype controls and were incubated at 4°C for 20 min. After permeabilization with Cytofix/Cytoperm (BD Biosciences), the cells were stained with 100 µL of an anti-alphavirus (cat. MAB8754, Merck) at 1:100 (vol/vol) diluted in Perm/Wash (BD Biosciences). After incubation, the cells were washed and stained with 100 µL of Alexa Fluor 488-conjugated goat anti-mouse IgG (Invitrogen) at 1:400 (v/v) in Perm/Wash solution. Finally, the cells were washed and recovered in 200 µL of PBS/1.5% paraformaldehyde. The cell suspensions were analysed by flow cytometry on a FACSCanto II instrument (BD Biosciences). Data were expressed as the means ± SEM. Statistical analyses were performed using PRISM (version 7.0; GraphPad, San Diego, USA). Significance was determined using a paired nonparametric test (Wilcoxon) among different stimulations in the same group. Values of *p* ≤ .05 were considered significant.

## Results

### Molecular prospecting and phylogenetic analyses

A total of 1,923 mosquitoes grouped into one hundred twenty-one pools were collected at Marilena municipality, Brazil, where an unknown arthritogenic disease outbreak was taking place (Table S1). Nine genera of mosquitoes were identified by morphological identification: *Aedes*, *Aedeomyia*, *Anopheles*, *Culex*, *Limatus*, *Mansonia*, *Psorophora*, *Sabethes*, and *Weyomyia*. Three out of 121 pools of mosquitoes analysed yielded positive results for alphavirus by RT-PCR. Two positive-pools (codes MS680 and MS681) had exclusively female mosquitoes (n = 21 and 9 mosquitoes per pool, respectively), while the third positive-pool (code MS364) contained 7 mosquitoes and was not characterized by gender. Forty-four out of the mosquito pools tested positive for flavivirus in a screening by a generic RT–PCR protocol [16]. Amplicons varying from 98 bp to 1167 bp were sequenced. These amplicons were identified as insect-specific flaviviruses (ISFs) using BLAST algorithm (available in: https://blast.ncbi.nlm.nih.gov/Blast.cgi). The alphavirus cDNA fragments, amplified from the mosquito pools, were identified via genomic sequencing of a 455-bp amplicon from the nonstructural protein 4 (nsP4) gene. Nucleotide alignment with sequences retrieved from GenBank showed by BLAST algorithm a 74% of nucleotide identity with a group of encephalitogenic viruses, such as VEEV, Western Equine Encephalitis (WEEV), and Eastern Equine Encephalitis (EEEV). The use of the combined protocols of Sánchez-Seco et al. [15] and Hermanns et al. [4] for the same mosquito pools resulted in the amplification of a larger fragment of 1,028 bp corresponding to the nsP4 region that displayed a 76% of similarity to the equine encephalitis complexes. This low score raised the possibility that the identified alphavirus was novel, since it met the criteria of the International Committee on Taxonomy of Viruses for a distinct species within an antigenic complex (minimum of a 21% divergence at nucleotide level).

The virus was successfully isolated from the MS680, MS681 and MS364 pools in C6/36 cells, which presented a mild cytopathic effect. The isolation was confirmed by RT–PCR and by indirect IFA and the identity of the three isolates was confirmed by sequencing of the RT–PCR amplicons. An insect-specific flavivirus was co-isolated with CAAV from the MS680 and MS364 pools. For this reason, the subsequent viral characterization was performed with the virus isolated from the MS681 pool. Viral titres in C6/36 cells ranged from 10^5^ ffu/mL on passage 1 to 10^7^ ffu/mL on passages 2 and 3.

Next generation sequencing (NGS) of the third passage of the isolated virus yielded the viral genome in a single contig containing 12,096 bp with an average coverage of 1,846×. Comparison of the complete genome sequence with the GenBank nucleotide database using BLASTn showed a 73% identity with equine encephalitis complexes, corroborating the previous results. The nucleotide sequence was deposited in the GenBank database under the accession number MK353339.

**Figure 2.**
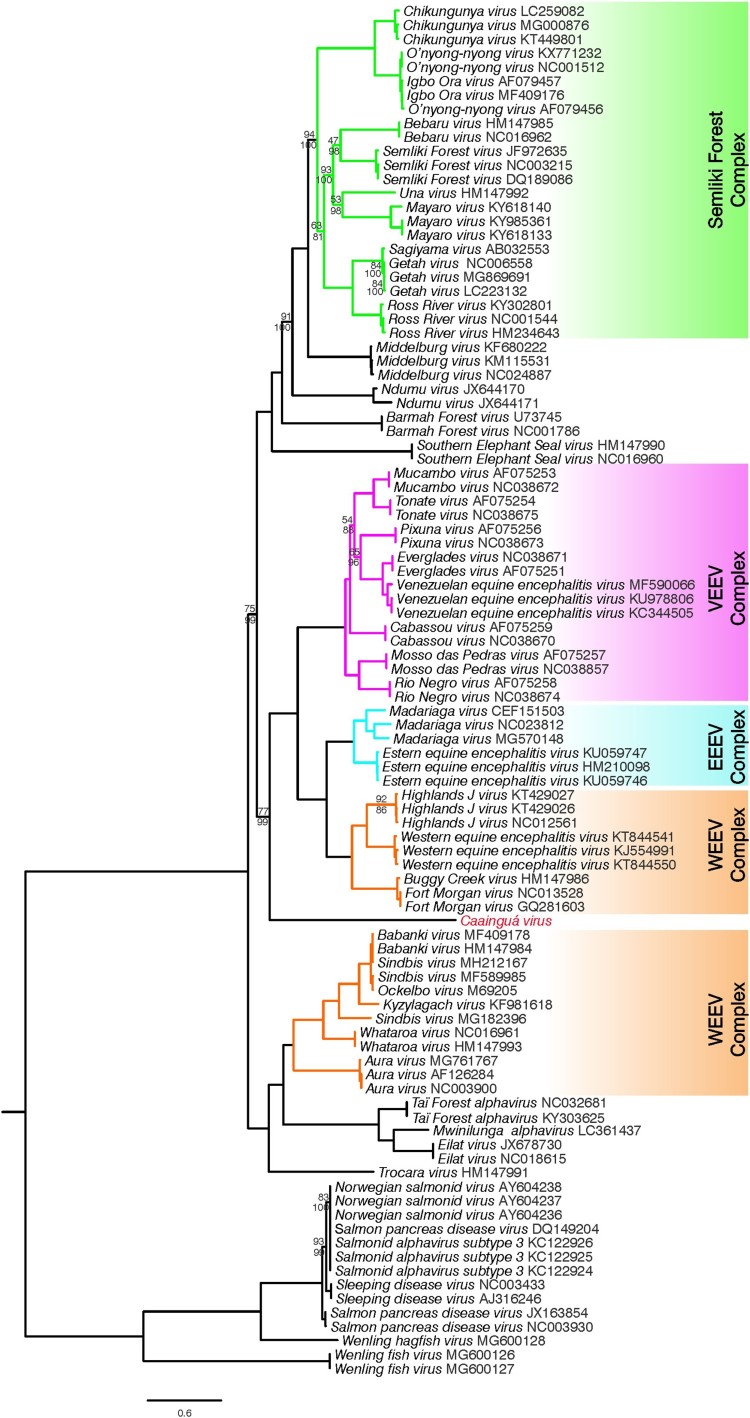
Maximum likelihood analysis of Caainguá virus incorporated with previously described alphaviruses and its variants based on nucleotide sequences of complete genomes. Bootstrap values over 95% were suppressed, those values lower than 95% are shown above the branches, and the related posterior probability values from Bayesian analysis are shown below the branches. The scale bar indicates the number of substitutions per site

ML and Bayesian methods were used to infer the phylogenetic relationship of the new isolated virus, hereafter referred as CAAV, within the genus *Alphavirus*. Three sequence alignments were analysed – (i) complete genomes; (ii) nonstructural protein (nsP) region; (iii) structural protein (sP) region – and both Bayesian and ML methods generated the same tree topologies when comparing the same alignment. Using the full genome analysis (Figure 2), CAAV was placed basal (bootstrap support of 77 and posterior probability of 99) to the encephalitogenic alphaviruses belonging to New World group which comprises VEEV, EEEV, and part of the WEEV complexes.

Phylogenetic trees generated from the nsP and sP genomic regions were compared to identify potential recombination events within CAAV genome (Figure S1). The use of this approach allowed for the detection of the already described recombinant origin of WEEV and some closely related viruses [27], which group together with VEEV and EEEV complexes when only nsP region is analysed but remain as a cluster with other species from the WEEV complex in the sP region tree. Overall, the nsP phylogeny was highly similar to that observed in the complete genome tree and differed only by the position of Aura virus (AURAV). CAAV clustering in the nsP tree (bootstrap support of 82) was consistent with the full genome tree, whereas in the sP analysis, it was placed basal to all encephalitis viruses (although the bootstrap support of 63), including the alphaviruses with unknown vertebrate hosts. In addition, more detailed analyses were performed to detect recombination using the GARD and bootscanning methods, but recombination breakpoints were not identified in CAAV. While our bootscanning specifications allowed the mosaic structure of WEEV to be reconstructed, CAAV did not clustered with high support within any of the described complexes or single alphavirus species in any analysed window (Figure S2).

DNA barcoding of mosquitoes allowed for precise species identification for one pool and an inaccurate identification to the other two. Sequences were checked using BLAST, and 854–872 bp fragments exhibited a 99% sequence identity for *Culex idottus* as the mosquito host in the MS681 pool and over 97% identity for multiple *Culex* species, such as *C. declarator*, *C. bidens*, *C. nigripalpus*, *C. mollis*, and *C. chidesteri* in the MS364 pool. Sequences analysis for MS680 pool showed 86–90% identity for different species of Sabethini.

### Transmission electron microscopy of the CAAV isolate

**Figure 3.**
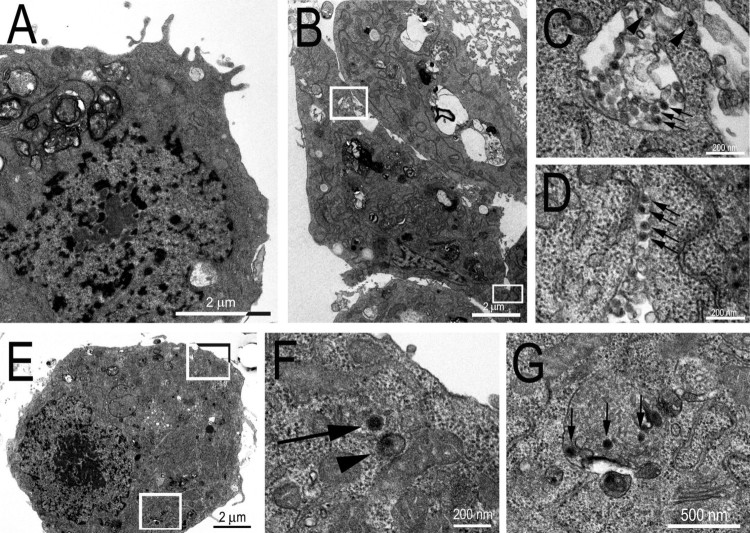
Transmission Electron Micrographs of C6/36 infected cells. (A) Mock cell. (B) CHIKV infected cell (positive control); (C) inset from (B) showing viral particles inside an intracellular compartment (arrows) and budding to the extracellular medium from the membrane (arrow heads); (D) inset from (B) showing viral particles in the extracellular medium between two cells. (E) CAAV infected cell; (F) inset from (E) showing CAAV particles frequently observed in the cytosol of infected cells associated with a membrane (arrowheads) or free (arrow); and (G) inset from (E) showing CAAV in intracellular compartments (arrows).

The use of transmission electron microscopy allowed us to observe that the cellular distribution of viral particles was similar in C6/36 cells infected with CAAV or CHIKV (used as a positive control, Figure 3). Scattered CAAV virions were observed in the cytosol and inside the intracellular compartments of infected cells, with virions appearing spherical in shape and measuring no more than 70 nm in diameter, like other alphaviruses. However, the frequency at which viral particles were observed in extracellular areas was higher for cells infected with CHIKV. In an attempt to enhance the amount of intracellular viral particles to better characterize the cellular distribution of CAAV virions, a higher multiplicity of infection (MOI – 10) and a longer period of infection (5 days post infection, d.p.i) were tested, but no significant enhancement of the number of particles was observed.

### Characterization of CAAV infection in different cell lines

**Figure 4.**
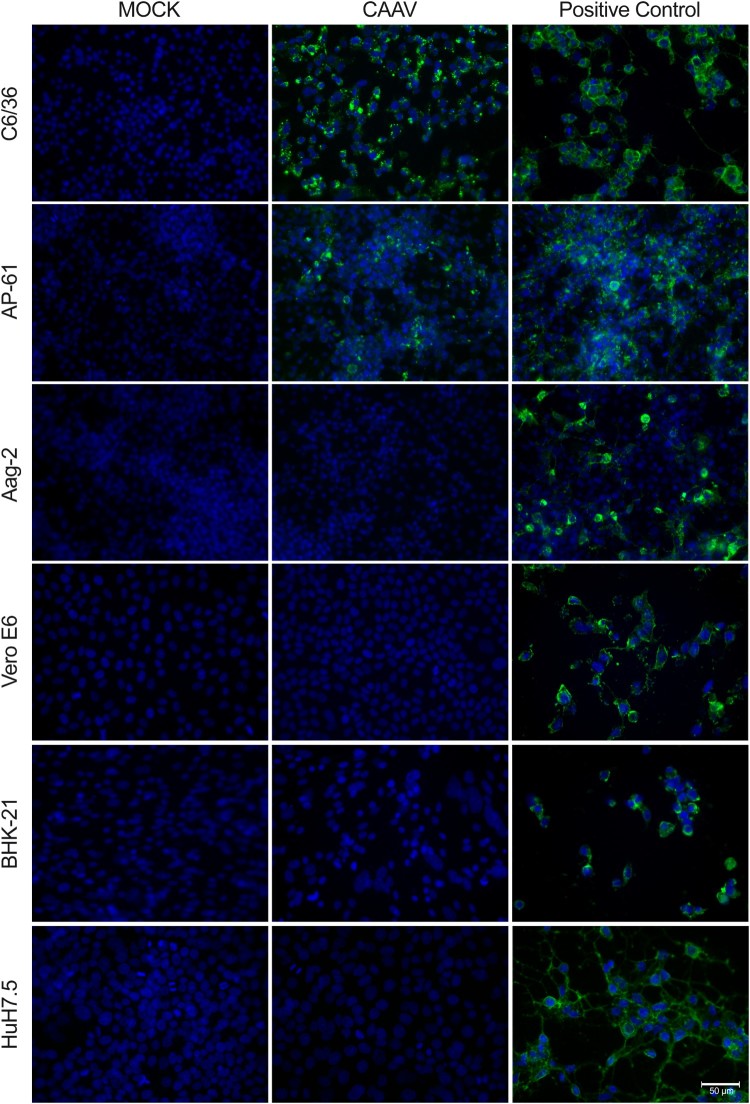
Infection of mosquito and vertebrate cells analysed by indirect immunofluorescence assay. Cells were infected by either CAAV or CHIKV (positive control) at an MOI of 1 for 72 h. Uninfected cells (Mock) were used as negative controls. The reaction was detected using an anti-alphavirus monoclonal antibody (cat. MAB8754, Merck) followed by Alexa-Fluor 488-conjugated anti-mouse IgG. Cell nuclei were stained with DAPI. Scale bar is 50 µm. Four additional vertebrate cells are shown in Supplementary Material Fig. S3.

Replication of CAAV in three different arthropod-derived cells lines was observed by IFA (Figure 4). *Aedes albopictus* C6/36 and *Aedes pseudoscutellaris* AP-61 cell lines were permissive to CAAV infection, but with distinct degrees. CAAV replication was more evident in C6/36 cells than in AP-61, differences that were not observed in the CHIKV infected controls. *Aedes aegypti* Aag2 cells were infected only by CHIKV (positive control).

Vertebrate-derived cell lines were also inoculated with CAAV, but no viral RNA transcription (data not shown) or envelope (E1) protein synthesis was detected in these samples (Figures 4 and S3). CHIKV and VEEV infections (positive controls) caused cytopathic effect 72 h post infection (h.p.i.) at an MOI of 1 in the cell lines tested. In contrast, CAAV did not induce morphological changes in any of the vertebrate-derived cell lines tested, even with a higher MOI, such as 10 (data not shown) after 72 h.p.i.

### CAAV infection on primary human cells

**Figure 5.**
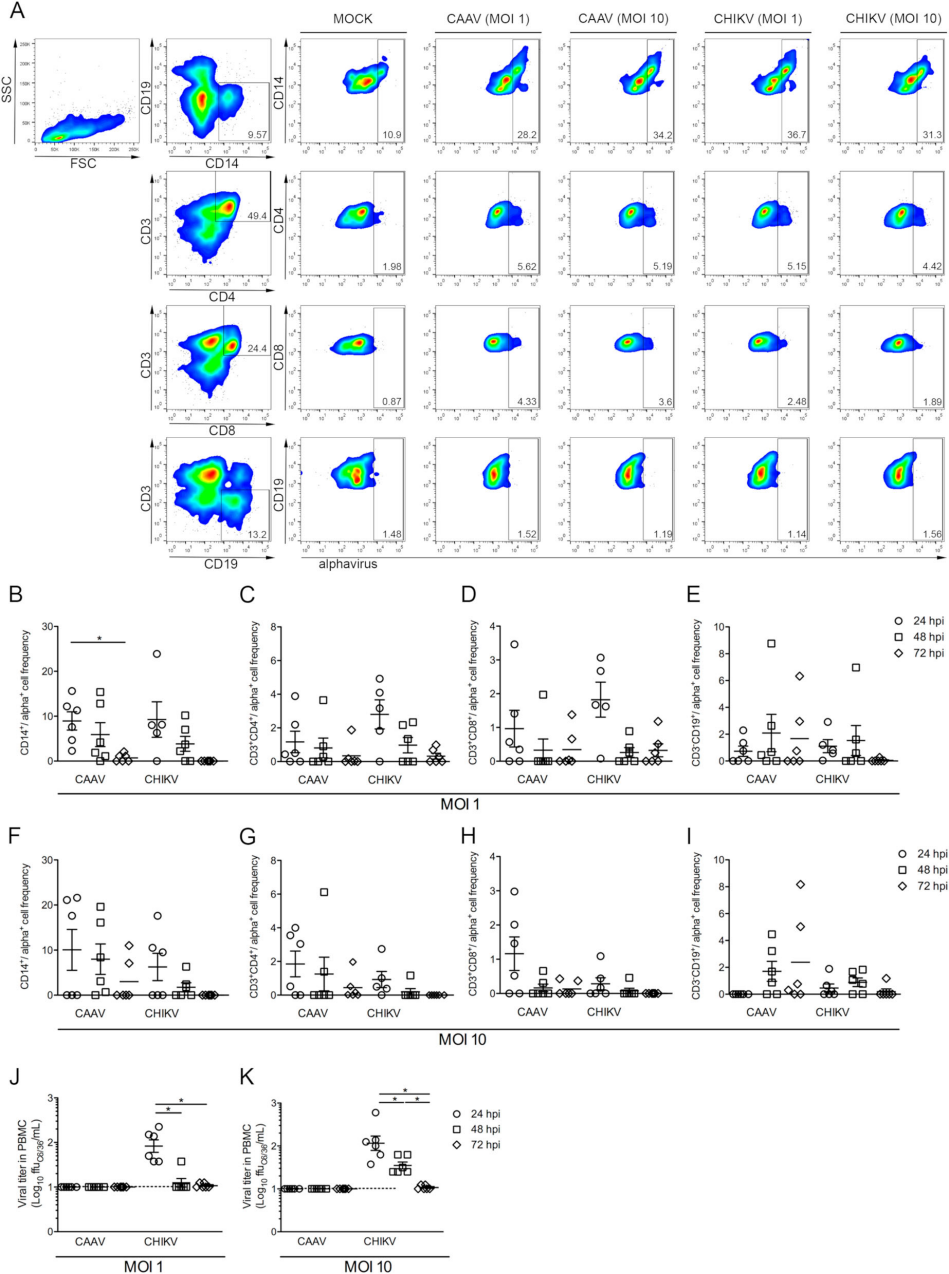
Infection of human mononuclear cells with CAAV**.** PBMCs from six healthy donors were infected with CAAV or CHIKV at an MOI of 1 or 10, and cells were analysed a 24, 48, and 72 h postinfection (h.p.i). (A) Representative flow cytometry density plot data showing the results for CD3^−^CD14^+^ (monocytes), CD3^+^CD4^+^ and CD3^+^CD8^+^ (T lymphocytes), and CD3^−^CD19^+^ (B lymphocytes) for Mock and infected (anti-alphavirus) cells 24 h.p.i. Bars represent the average frequencies of infection. Normalized infection frequency was expressed after subtracting the autofluorescence signal from mock samples. (B and F) CD3^−^CD14^+^ frequency of infection, (C and G) CD3^+^CD4^+^ frequency of infection, (D and H) CD3^+^CD8^+^ frequency of infection, (E and I) CD3^−^CD19^+^ frequency of infection. Viral particles were quantified in the PBMC supernatant using a focus-forming assay in C6/36 cells (ffu/mL). (J) Viral titres in PBMCs infected with virus at an MOI of 1, (K) viral titres in PBMCs infected with virus at an MOI of 10. *P ≤ .05.

To simulate a viral-human transmission cycle and test whether human cells were susceptible to CAAV infection, we infected PBMCs with CAAV and CHIKV (positive control) at two different MOI (1 and 10) and performed a flow cytometry analysis 24, 48 and 72 h.p.i. to evaluate different cell populations (Figure 5(A–I)). Monocytes (CD14^+^) were the primary cell population infected by both viruses, peaking at 24 h.p.i. (Figure 5(B,F)), followed by helper T lymphocytes (CD3^+^CD4^+^) (Figure 5(C,G)), and cytotoxic T lymphocytes (CD3^+^CD8^+^) (Figure 5(D,H)). A reduction in the frequency of positive cells during the infection kinetics was observed, which was significant for monocytes infected with CAAV when comparing the 24 versus 72 h.p.i. timepoints. Interestingly, the frequency of CAAV infected B lymphocytes (CD3^−^CD19^+^) increased at 48 and 72 h.p.i. (*p* > .05), which was not observed when cells were infected with CHIKV (Figure 5(E,I)).

We next investigated whether human mononuclear cells support CAAV replication and produce viable viral particles using a focus-forming assay (Figure 5(J,K)). Although positive cells were observed by flow cytometry analysis by the presence of cytoplasmic alphavirus envelope protein, viral titration in cell supernatants indicated that no cell-free viral particles were produced. In contrast, CHIKV particles were detected in supernatants at 24 and 48 h.p.i., but a drastic reduction was observed at later time points.

## Discussion

In this study, we describe a new alphavirus, named CAAV, isolated from *Culex* mosquitoes, which were collected during an outbreak of an unknown arthritogenic disease in Marilena municipality, Southern Brazil. As patient symptoms were compatible with classical alphavirus symptomatology, we assessed whether this new isolate could be associated with disease in vertebrates, especially because phylogenetic analysis demonstrated that CAAV is placed basally in the clade of the encephalitic alphavirus complex. However, no irrefutable evidence links this new alphavirus to the human cases in Marilena. Although the results indicate the discovery of a new insect-specific alphavirus, the observed infection of PBMCs indicates that further investigation is needed to clarify the CAAV transmission cycle in nature.

We present a complete phylogeny analysis of alphavirus based on all available complete viral genomes. Phylogenetic analysis placed CAAV basal to the New World encephalitic alphavirus complexes in both the complete genome (Figure 2), and nsP trees (Figure S1, right panel). This group is separated from those viruses belonging to the WEEV complex that originated in the Old World, such as Sindbis (SIN)-like viruses. In our analysis, SIN-like viruses grouped with AURAV first isolated in Brazil [28] and with alphaviruses recently discovered in Israel, Ivory Coast, and Zambia for which vertebrate hosts are unknown [3–5].

Despite the low bootstrap support, the CAAV translocation to a deeper node of the encephalitic alphavirus clade in the sP tree (left of Figure S1), could suggest a recombinant origin, similar to that described for WEEV, Highlands J virus (HJV), Fort Morgan virus (FMV), and the variant Buggy Creek virus (BCRV) [27]. These recombinant viruses grouped with the SIN-like clade in the sP phylogeny, consistent with the evolutionary origin of the envelope proteins of the recombinant ancestor from a SIN-like ancestor. A recombination event combined the envelope protein gene from a SIN-like virus and the remaining genes from an EEEV-like ancestor, resulting in the ancestral WEEV, HJV, FMV, and BCRV [27,29,30]. However, the recombination analyses performed did not detect a recombination signal in CAAV (Figure S2). Furthermore, the use of a bootscanning approach with window size of 1,000 bp showed the uniqueness of CAAV, since it did not cluster with high bootstrap support with any of the previously described alphaviruses or its serological complexes. The phylogenetic placement of CAAV is of interest, since it reinforces the most parsimonious scenario for the evolutionary origin of the encephalitic alphavirus clades on the American continent described by Weaver et al. [30]. CAAV is included among the members of the VEEV, EEEV, and WEEV complexes that were originally New World viruses, excluding the SINV-like group.

However, based on the results of this study, the origin of the alphavirus genus remains unclear, with the alphaviruses emerging as arthritogenic viruses in the Old World being posteriorly introduced in the New World, or vice versa, emerging in the New World as encephalitic viruses and spreading to the Old World [31]. A marine origin of alphaviruses was previously proposed by Forrester et al. [32] which suggested a Pacific emergence of alphaviruses from marine to terrestrial vertebrate hosts and to mosquito vectors, although they could not identify a robust placement for the Southern elephant seal virus (SESV) isolated from lice [33]. Nevertheless, this hypothesis is not supported by our results. In the present study, in both Bayesian and ML analyses of complete genomes and fractionated open reading frames, SESV was placed basal to the Semiliki Forest complex along with Middelburg virus, Ndumu virus, and Barmah Forest virus (Figures 2 and S1). The arthropod-borne clade remains in an independent clade relative to the fish alphavirus clade, including salmonids, by horizontal transmission [34], as well as the recently discovered virus that infect frogfishes and hagfishes sampled between the South China Sea and Yellow Sea [6]. Furthermore, many discoveries of RNA viruses in salt water are expected [35] and will likely better address the origin of the genus in the future.

Emerging viral diseases can arise from a number of scenarios: genetic changes (evolution), including the genetic adaptability of RNA arboviruses to new hosts; the spread of pathogenic organisms to new geographic areas and/or contact with new potentially susceptible host populations; ecologic changes that increase the exposure of naive host populations to the pathogenic agent; and environmental sources that may harbour new or unusual infectious agents [36–39]. Surveillance strategies often involve combating emerging arboviruses as they occur (primary preventions) using epidemiological data to mitigate the damage. However, the re-emergence of arboviruses has increased in the last decades, with the major driving forces being the increase in unplanned urbanization, mosquito adaptation to the urban environment, intensive growth of global transportation systems, and failure to control the density of mosquito populations [for an overview see ref. 40]. These factors have culminated, for example, in the emergence of three new arboviruses in the Americas: WNV in 1999 [41,42], CHIKV in 2013 [8,9], and Zika virus (ZIKV) in 2015 [43,44].

The sensitivity of different cell lines to viral infection can differ considerably [45,46]. The C6/36 cell line was the most permissive to CAAV compared to other assayed mosquito cell lines, as shown in Figure 4. The AP-61 cell line was less permissive to CAAV infection than C6/36 cells, even at a high MOI (of 10), whereas the Aag2 cell line was refractory to the infection, unlike CHIKV. Each of these cell lines was derived from a different species of mosquitoes and have been shown to have different susceptibilities to arbovirus infection [47]. C6/36 cells were previously described to have a dysfunctional RNAi-mediated antiviral response [48], making it a good model to perform viral isolation, whereas Aag2 cells are frequently used as an immune system model against viral infections due to its effective Dicer-2 pathway [49]. Interestingly, inhibition of RNAi pathway was shown to potentially favour alphavirus replication in both cellular models and in *A. aegypti* mosquitoes [48–50]. The electron microscopy images show that fewer CAAV particles were produced in infected C6/36 cells 72 h.p.i. compared to CHIKV, for which numerous particles could be observed budding from cell membranes (Figure 3). Although viral particles were observed in cytoplasmic vesicular compartments, there is currently a debate regarding whether such particles result from the internal budding of endocytosed viruses [51].

*In vitro* cell line models are broadly used to understand mechanisms of viral pathogenesis and to predict whether a virus is capable of infecting vertebrate hosts. We did not detect CAAV envelope protein by IFA in any of the seven vertebrate cell lines tested, even when using interferon type 1 deficient cell lines, such as Vero E6 and BHK-21. Studies using chimeric viruses showed that this restriction of productive mammalian cell infection can be related to both structural (at the entry level) and nonstructural proteins (nonfunctional replicative complexes are formed) [52]. Viral fitness can also vary as a function of the cell substrate [53], and a putative restriction of productive infection in vertebrate cells after successive C6/36 passages was previously reported [54]. Interestingly, we demonstrated that primary human mononuclear cells are susceptible to CAAV infection *in vitro*, since E protein was detected intracellularly (Figure 5). Other alphaviruses, such as Ross River virus (RRV), CHIKV, SINV and MAYV have been reported to infect and replicate in mouse macrophages [55–58], and CHIKV infection was detected in human blood monocytes from acutely infected patients [59]. In this study, we showed that monocytes were the primary cell population targeted by CAAV, followed by CD4^+^ and CD8^+^ T lymphocytes. Similar results were also obtained using CHIKV. These results are of particular interest, since these are the main cells involved in inflammatory cell infiltration during the development of arthralgia in animal models [60]. However, unlike CHIKV, CAAV did not result in a productive infection (viable viral particles were not detected in supernatants). Interferon-inducible hosts factors, such as tetherin, can restrict the ability of enveloped viruses to bud out from infected cells, and this protein is notably well expressed in hepatocytes, monocytes and activated T lymphocytes [61]. Though, it cannot be discarded that the observed reduction in the frequency of infected cells and viral titres could be due to the death of infected cells. The susceptibility of different cells to CHIKV infection is controversial. Sourisseau et al. [62] showed that most of the epithelial and fibroblasts cell lines tested were susceptible to viral infection and replication, but lymphoid and monocytic cell lines, as well as primary monocytes and lymphocytes and monocyte-derived dendritic cells were refractory to CHIKV [62]. Moreover, viral replication exerts a cytopathic effect that was associated with apoptosis of infected cells [62].

Unexpectedly, envelope protein was gradually detected in B lymphocytes, peaking at 72 h.p.i., indicating the productive protein synthesis of CAAV. On the other hand, this was not observed with CHIKV, reinforcing that the E protein synthesis is not a spurious phenomenon. We are tempting to speculate that one possible mechanism for the later B lymphocyte infection is the well described cell-to-cell transmission of viruses [52]. Since free viral particles were not detected in PBMC supernatant, but the monocyte flow cytometry analysis showed the intracellular presence of the CAAV, we speculate that infected monocytes could be the source of virus that were transmitted via long intercellular extensions, delivering CAAV to the B lymphocytes. Intercellular extensions that provide cell-to-cell contact have been reported for different alphaviruses, such as SINV, SFV, CHIKV [63–65]. Brown et al. [52] suggested that the interface between long extension from the infected cell favours the internalization of the virus by an uninfected cell and protects the virus from neutralizing antibodies.

One factor that might challenge viral detection is low viremia titres, which can broadly vary depending on the etiologic agent and can range from <10^2^ for VEEV [66] to 10^8^ pfu/mL during the peak of CHIKV disease [67]. Thus, the unsuccessful detection of CAAV from Marilenás human samples could be due to low viremia titre or to other factors, such as inappropriate conditions used to store samples prior to their shipment to the Reference Laboratory.

Despite the data concerning the ability of CAAV infect vertebrate cell lines are controversial, we emphasize that, to date, Eilat virus, Mwinilunga alphavirus and Taï Forest alphavirus are the insect-specific Alphavirus described and placed basally in the Western Equine Encephalitis Complex [3–5,68,69]. Thus, the description of a novel Alphavirus placed in the New World clade can improve the understanding about the evolution of Alphaviruses, especially when we consider the hypothesis that arboviruses might arise from insect-specific viruses.

Summing up, CAAV is an alphavirus closely related to the equine encephalitis complexes, that was isolated from mosquitoes and can be internalized by primary human cells. Although cell line experiments may suggest CAAV as a new insect-specific Alphavirus, the results regarding CAAV infection on PBMC are intriguing. Additional studies are necessary to investigate the CAAV cycle and its relevance to public health.

## Etymology

Caainguá virus (CAAV) is a tribute to the indigenous tribe of the Guarani Indians that inhabited the region. Caainguá is a variant of the name, which in the Tupi-Guarani language means “who lives in the wood/jungle.”

## Supplementary Material

Supplemental Material
