# Supplementary figures and images for: Identification of a novel alphavirus related to the encephalitis complexes circulating in southern Brazil
Source: Emerg Microbes Infect. 2019 Jun 25;8(1):920–33. doi: 10.1080/22221751.2019.1632152 (PMC6598490; doi:10.1080/22221751.2019.1632152)

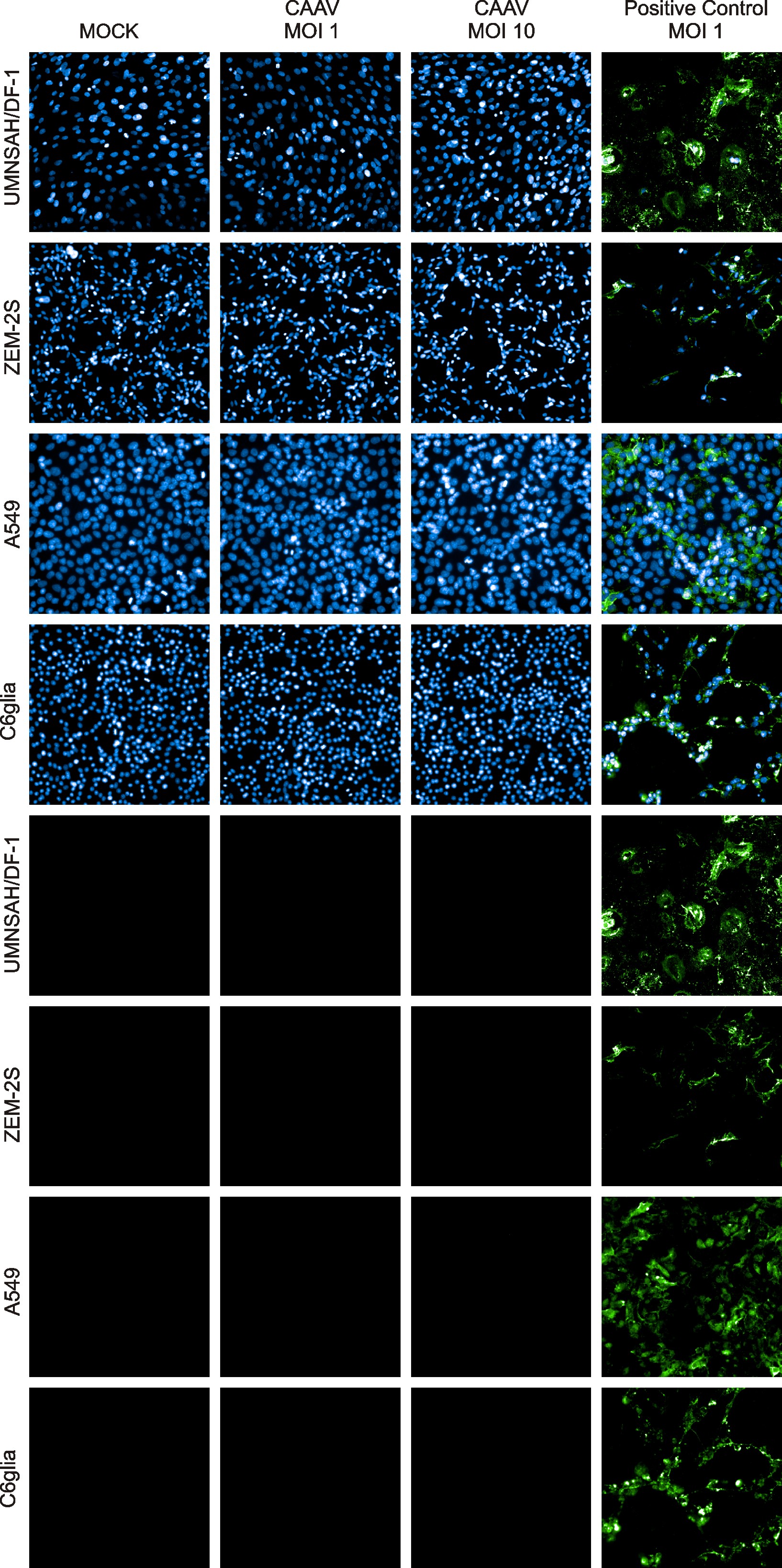

Supplement: Supplemental Material [file TEMI_A_1632152_SM6791.zip › Supplementary Material/temi-2019-0026-20190612190150/doc/Fig S3.jpg]

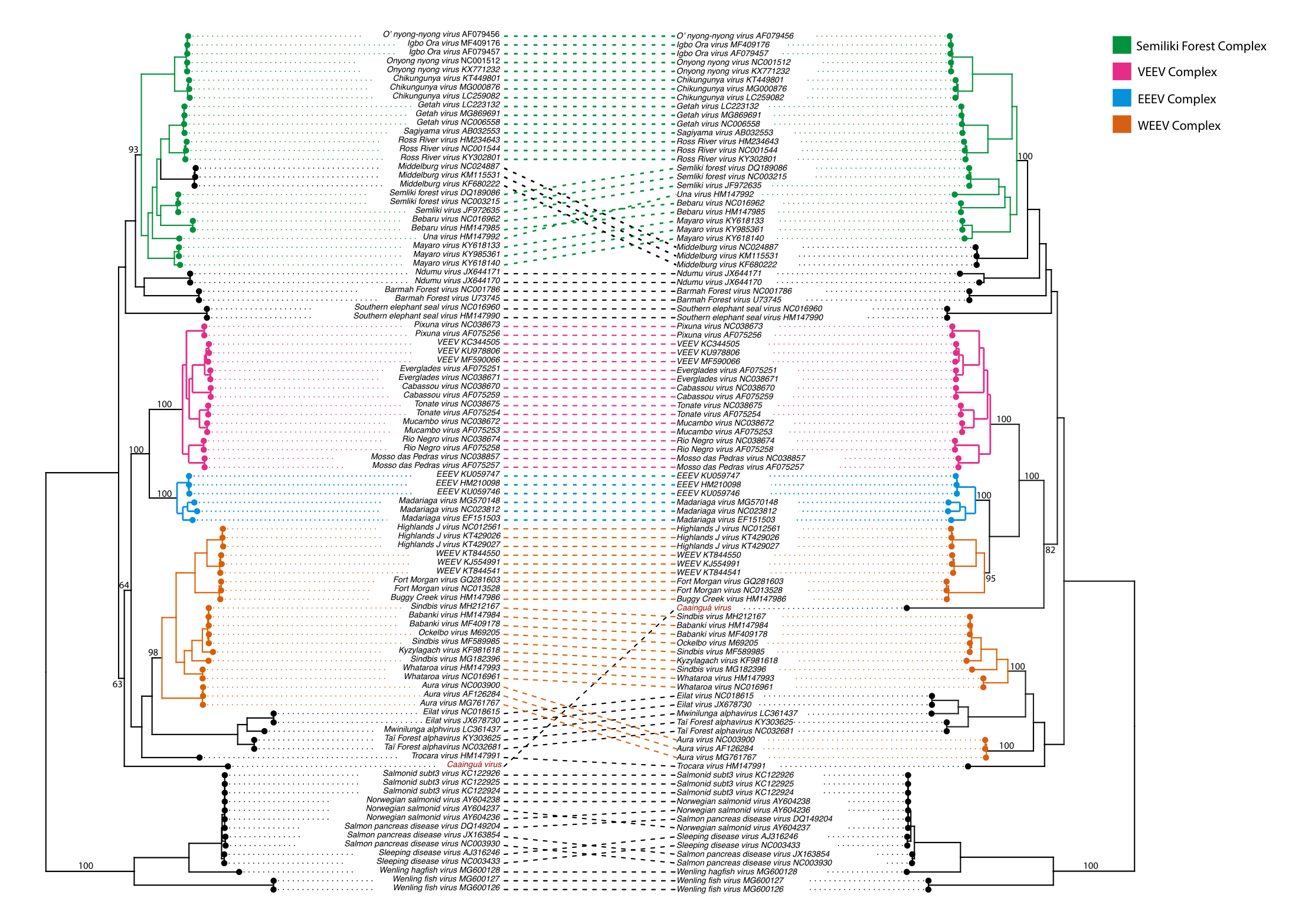

Supplement: Supplemental Material [file TEMI_A_1632152_SM6791.zip › Supplementary Material/temi-2019-0026-20190612190150/doc/Figure S1 final.tiff]

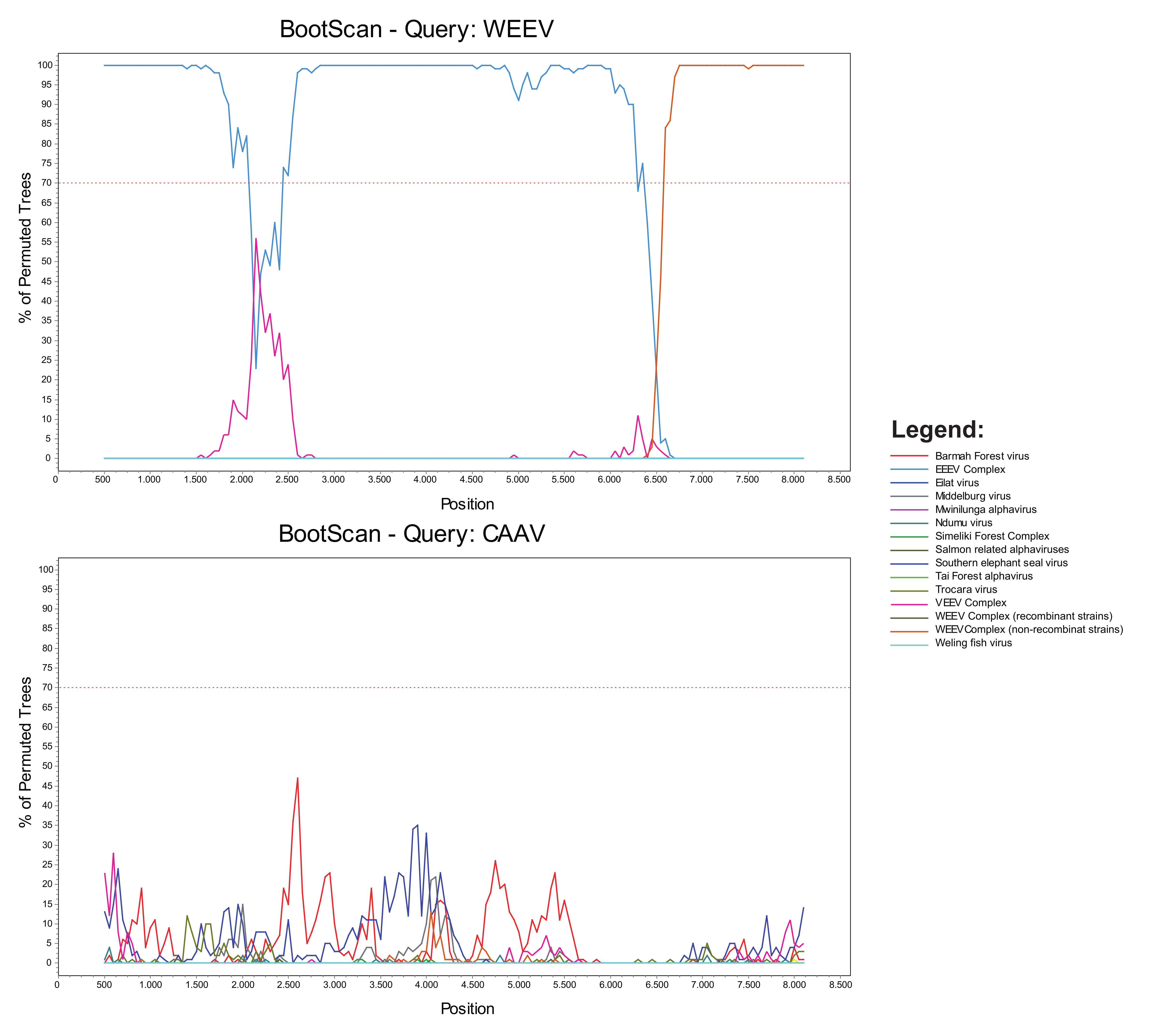

Supplement: Supplemental Material [file TEMI_A_1632152_SM6791.zip › Supplementary Material/temi-2019-0026-20190612190150/doc/Figure S2.tiff]
